# Long Non-coding RNAs in Cancer: Implications for Diagnosis, Prognosis, and Therapy

**DOI:** 10.3389/fmed.2020.612393

**Published:** 2020-11-30

**Authors:** Yuchen Qian, Lei Shi, Zhong Luo

**Affiliations:** School of Life Sciences, Chongqing University, Chongqing, China

**Keywords:** long non-coding RNAs, cancer, biomarker, diagnosis, prognosis, therapy

## Abstract

Long non-coding RNAs (lncRNAs) are major components of cellular transcripts that are arising as important players in various biological pathways. They have received extensive attention in recent years, regarded to be involved in both developmental processes and various diseases. Due to their specific expression and functional diversity in a variety of cancers, lncRNAs have promising applications in cancer diagnosis, prognosis and therapy. Studies have shown that lncRNAs with high specificity and accuracy have the potential to become biomarkers in cancers. LncRNAs can be noninvasively extracted from body fluids, tissues and cells, and can be used as independent or auxiliary biomarkers to improve the accuracy of diagnosis or prognosis. Currently, the most well-recognized lncRNA is PCA3, which has been approved for use in the diagnosis of prostate cancer. Moreover, the underlying mechanisms of lncRNAs were explored as therapeutic targets, which have been investigated in clinical trials of several cancers. In this review, we presented a compilation of recent publications, clinical trials and patents, addressing the potential of lncRNAs that could be considered as biomarkers or therapeutic targets, with the hopes of providing promised implications for future cancer therapy.

## Introduction

Cancer is a life-threating disease with rising morbidity and mortality (1). Despite tremendous progress made in recent years, there are still a number of issues in cancer treatment that need improvements, such as delayed diagnosis and poor prognosis (2). Most tumor biomarkers or therapeutic targets currently in clinical use are proteins. However, only 2% of human genome is translated into proteins. Therefore, we may need to focus more on non-coding regions, where more cancer mutations occur than in coding regions (2, 3). In recent years, long non-coding RNAs (lncRNAs), which occupy the majority of non-coding RNAs (ncRNAs), are hotspots in cancer research. Due to the large number of lncRNAs, with an estimate of 102,000, lncRNA-based research holds great promise in cancer treatment (3, 4).

LncRNAs are non-coding transcripts with more than 200 nucleotides in length, and most of them remain in the nucleus after transcription (5, 6). Due to their low expression levels, lncRNAs were initially considered to be transcription noise. With better understanding, lncRNAs are found to be involved in transcriptional and post-transcriptional regulation, through interactions with DNA, RNA or proteins (6). LncRNAs promote or inhibit the formation of transcription loops, and recruit or block regulators, regulating gene transcription (7–9). Besides, lncRNAs also regulate mRNA splicing and act as precursors to other ncRNAs, such as microRNAs (miRNAs) (10). LncRNAs function as oncogenes or tumor suppressors, taking part in various signaling pathways (11). Of note, by analyzing the expression of lncRNAs in peripheral blood, urine sediments or tissue samples, a series of lncRNAs were identified with great promise as auxiliary or independent biomarkers in cancer diagnosis and prognosis (12).

There are currently few biomarkers or therapeutic agents targeting lncRNAs. Prostate cancer antigen 3 (PCA3), an early diagnostic biomarker for prostate cancer (PCa), is the first and only approved lncRNA for clinical use at this time (13). There are also some lncRNAs undergoing clinical trials or having been patented, which we will discuss in more details below. Moreover, other research of lncRNA-based drug discoveries, including UBE3A-ATS in Angelman syndrome, SCN1ANAT in Dravet syndrome and SMN-AS1 in spinal muscular atrophy, also illustrate the potential of lncRNAs (14–17).

In this review, we discuss the mechanisms by which lncRNAs function. A thorough understanding of these mechanisms is critical for the development of anti-tumor drugs. We next summarize a collection of recent publications, clinical trials, and patents and also discuss the potential of lncRNAs that could be considered as biomarkers or therapeutic targets in cancer diagnosis, prognosis and treatment.

## Mechanisms of LncRNAs

**Figure 1:**
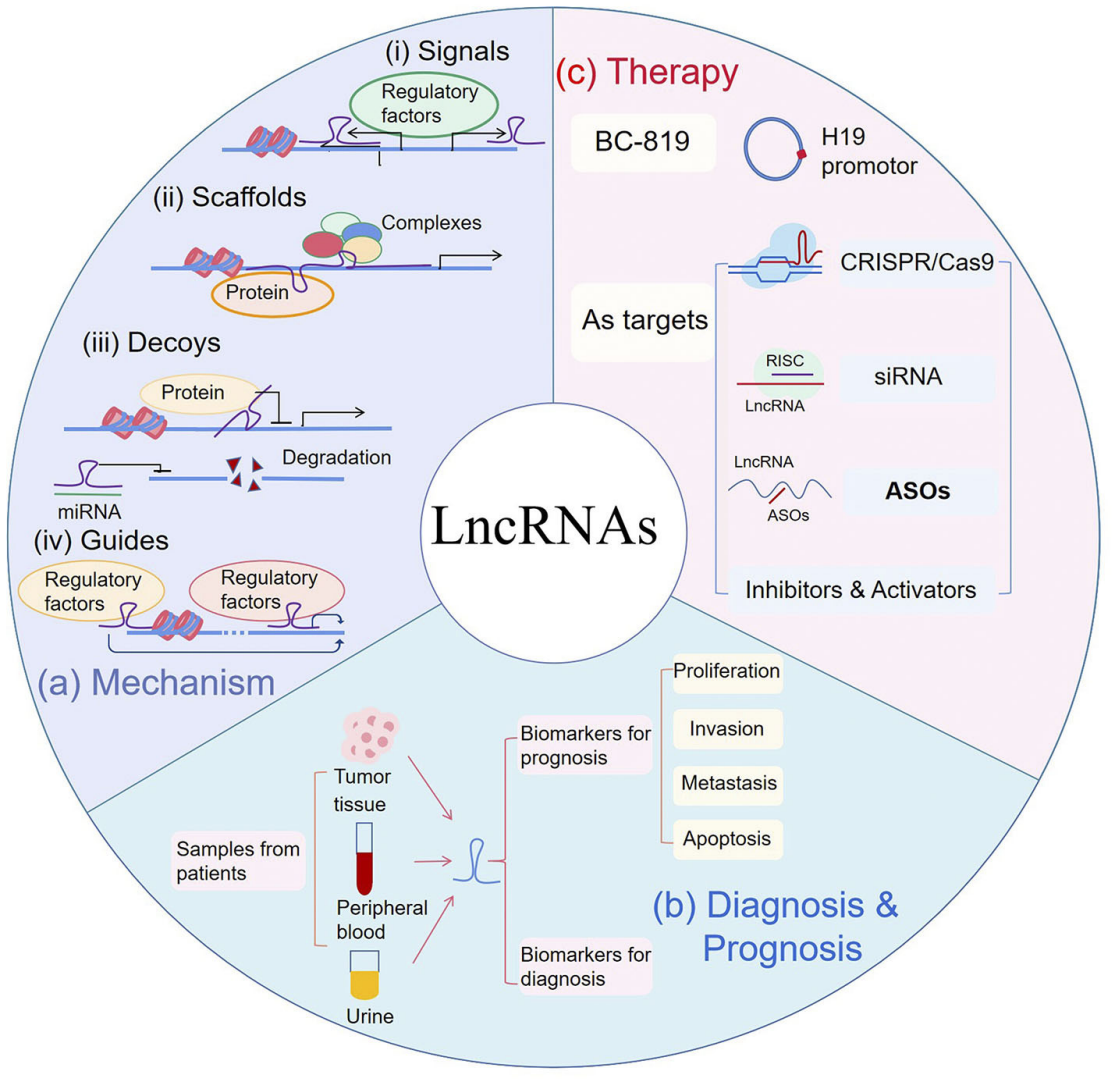
**(a)** Mechanism: (i) LncRNAs can serve as signals to reflect the activity of pathways or developmental status. (ii) LncRNAs act as scaffolds by recruiting proteins to regulate gene expression. (iii) LncRNAs can be used as decoys to block activities of proteins and can also bind to microRNA (miRNA) to inhibit miRNA-induced degradation. (iv) As guides, lncRNAs recruit transcription regulators to specific sites. **(b)** Diagnosis & Prognosis: As biomarkers for cancer diagnosis and prognosis, lncRNAs can be extracted from tumor tissues, peripheral blood and urine samples of patients. In prognosis, they are correlated to patient's proliferation, metastasis, invasion or survival. **(c)** Therapy: BC-819, fused with H19 promotor and Diphtheria toxin gene, was used in phase 2 clinical trial. Gene editing such as CRISPR/Cas9, small interfering RNA (siRNA) and antisense oligonucleotides (ASOs), were used to silence targeted lncRNAs. RISC RNA-induced silencing complex.

Understanding how lncRNAs work is critical to know how they cause diseases such as cancers, and therefore to their potential applications in cancer treatment. Based on current studies, lncRNAs are implicated in many intracellular molecular interaction networks. The levels of their expression are regulated by many factors, and they are also involved in complex networks as regulatory factors. The myriad mechanisms behind these complex regulations can be summarized in four ways, including signal, scaffold, decoy, and guide (Figure 1) (5, 18, 19).

- Some lncRNAs are expressed at different levels in various cell states. Thus, they can be turned into signals, serving as indicators to reflect development or disease status (18, 20). For example, Xist, which is typically transcribed by the inactive X chromosome, can be used to indicate X chromosome inactivation (21, 22).
- LncRNAs can bind proteins and act as scaffolds to assist in the assembly of regulatory complexes (23). In this way, HOTAIR interacts with polycomb repressive complex 2 (PRC2) to recruit EZH2 to promote H3K27 trimethylation or LSD1 to demethylate H3K4me2 (23, 24).
- As decoys, lncRNAs regulate gene expression by preventing the binding of transcription regulators (19, 25). For example, p53-dependent PANDA inhibits proptosis by directly sequestering of NF-YA (26). In addition, as competing endogenous RNAs (ceRNAs), lncRNAs also bind miRNAs and prevent RNA degradation (27). This is common in cancers. H19 acts as ceRNAs both for miR-17-5P in thyroid cancer and for miR-152 in breast cancer (27, 28).
- LncRNAs can also guide the transcription factors to specific sites (29). In this way, MEG3 guides PRC2 and forms a complex with DNA (30). It is noteworthy that each type is not mutually exclusive, and an individual lncRNA may have one or more of these functions (20).

## LncRNAs in Cancer Diagnosis

Some lncRNAs are highly tissue specific and abnormally expressed in cancer, which can be extracted noninvasively from the circulation (31, 32). These features render them potential candidates for cancer diagnosis (Table 1). The one most well-recognized is PCA3, a biomarker for early diagnosis of prostate cancer (PCa) (67). As mentioned above, it has been approved in clinical use (13). The diagnosis of PCa currently relies on the elevation of serum prostate-specific antigen (PSA) (12). However, its specificity in discriminating benign and malignant tumors is low, which may lead to over-diagnosis in low-risk patients (68). PSA is a good predictor when its level is above 10 ng/ml. However, in the gray area of PSA, some auxiliary indicators are still needed to improve diagnostic accuracy (12).

**Table 1:** LncRNAs as diagnostic or prognostic biomarkers.

**Cancer^**a**^**	**LncRNAs**	**Expression**	**Applications**	**References^**b**^**
Prostate	PCA3	Up	Diagnosis	(33–35), Approved
	MALAT1	Up	Diagnosis	(36), CN104498495
	LincRNA-p21	Up	Diagnosis	(37)
Bladder	PTENP1	Down	Diagnosis	(38)
	UCA1	Up	Diagnosis	(39)
	SPRY4-IT1	Up	Diagnosis	(40)
	HOTAIR	Up	Prognosis	(41)
Breast	H19	Up	Diagnosis	(42, 43)
	MALAT1	Up/Down^c^	Prognosis	(44, 45)
Colorectal	CCAT1	Up	Diagnosis	(46), NCT04269746, US20110097271A1
	CCAT2	Up	Prognosis	(47, 48)
	MALAT1	Up	Prognosis	(49)
	MEG3	Down	Prognosis	(50)
	HOTAIR	Up	Prognosis	(51, 52)
Gastric	HOTAIR	Up	Diagnosis	(53, 54), CN105586399A
	HOTAIR	Up	Prognosis	(55–57)
	MALAT1	Up	Diagnosis	(58), CN105586399A
	MALAT1	Up	Prognosis	(59)
Liver	MALAT1	Up	Prognosis	(60–62)
	H19	Up	Diagnosis	(42), CN105132559
Esophageal	CCAT2	Up	Prognosis	(48)
	PCAT1	Up	Prognosis	(63)
Glioma	CASC2	Down	Diagnosis	(64), CN103993088A
	CRNDE	Up	Prognosis	(65), CN103966339A
Thyroid	HOTAIR	Up	Diagnosis	(66), NCT03469544

a *The cancer types to which the indicated tissue corresponds*.

b *The accession numbers of clinical trials or patents were list as follows*.

c *The expression of MALAT1 in breast cancer is still controversial as described in the text*.

Urine PCA3 diagnosis is not only highly sensitive (58–82%) but also has excellent specificity (59–76%) (33). PCA3 is up-regulated 60 to 100-folds in more than 95% of PCa specimens (18, 33). It is noteworthy that PCA3-based assays are still effective when cancer cells make up <10% of the examined sample (33). PCA3 silence leads to an increase in the expression of E-cadherin, Claudin-3, and Keratin-18, while a decrease of Vimentin. The association between PCA3 and these traditional protein biomarkers provides more support for its application as diagnostic marker (34).

MALAT1 can be used as an auxiliary biomarker to improve the accuracy of early diagnosis, especially in the gray area of PSA. Its diagnostic accuracy is higher than the previous index, free/total PSA ratio (12, 69). In fact, one of the MALAT1 assay has been patented in PCa diagnosis (CN104498495).

H19 is another lncRNA with high diagnostic sensitivity and specificity. In breast cancer, patient plasma H19 levels were elevated, with an sensitivity of 0.81 (AUC, area under the curve), higher than traditional diagnostic biomarkers (42). H19 is related to many important miRNAs in the network of cancer-related functions (70). It is a precursor of miR-675 which has downstream targets like c-CbI, CbI-b and Igf1r (71, 72). H19/miR-675 also causes the activation of EGRF and c-Met, which leads to the sustained activation of Akt and Erk (43). Besides, H19 acts as a ceRNA for Let-7 to maintain the activation of breast cancer stem cells (73). The potential of miRNAs like miR-675 and Let-7 as biomarkers has been reported in cancers, which implicate the diagnostic potential of H19 (74). In gastric cancer (GC), H19 had a high diagnostic ability with an AUC of 0.838. A patent has been filed for gastric cancer diagnosis with HOTAIR and MALAT1 (CN105586399A), showing possible applications. Notably, single nucleotide polymorphisms (SNPs) of H19 are used to predict the risk of cancer, such as rs2839698 and rs2107425 genotypes are found to be related to decreased risk of bladder cancer (75).

LncRNAs are usually aberrantly expressed and can be extracted noninvasively from the circulation (32). Although some lncRNAs overlap in various cancer, ~60% of these abnormally expressed lncRNAs are cancer type-specific (31). Recently, the diagnostic potential of some lncRNAs has been implicated by clinical trials (76). One report have shown that UCA1 was sensitive for bladder cancer, especially in patients with superficial G2-G3 (77). Another clinical trial is underway to explore the possible application of CCAT1 in CRC (NCT04269746). Moreover, lncRNAs can not only be used as an independent biomarkers, but also can be combined with other lncRNAs or proteins to improve the sensitivity and accuracy of diagnosis (76).

## LncRNAs in Cancer Prognosis

The expression of lncRNAs in cancer correlates with overall survival (OS), metastasis, tumor stage or grade, thus can potentially serve as markers for prognosis (Table 1).

HOTAIR is transcribed from HOXC locus located at 12q13.13 (78). It has been proved to be poor prognostic indicators of various cancers. Bladder transitional cell carcinoma (TCC) patients with high HOTAIR have lower overall survival, and positively associated with histological grade (41).

An analysis of a large cohort containing 300 samples showed that the increased expression level of HOTAIR in GC tissues was correlated with peritoneal diffusion (55). In addition, in diffuse GC, tissues with a high HOTAIR level showed more venous infiltration and poorer overall survival (56). Besides, HOTAIR is involved in tumorigenicity in pancreatic cancer and can also cause CRC proliferation and metastasis mediated by PCR2 complex (51, 52).

MALAT1, located on chromosome 11q13.1, is dysregulated in many cancers (49). A cohort analysis of 169 patients showed that patients with high MALAT1 expression levels had a worse prognosis than the normal group (79). Moreover, upregulated MALAT1 is closely related to hepatocellular carcinoma (HCC) progression, and can be an independent biomarker for recurrence after liver transplantation (80). However, the roles of MALAT1 in breast cancer are still controversial. In previous reports, MALAT1 acts as a ceRNA for miR-1/CDC42 axis to enhance cell migration and invasion (44). On the contrary, Kim et al. reported that MALAT1 acted as a metastasis suppressor by preventing the binding of transcription factor TEAD and its co-activator YAP (45). In addition, MALAT1 knockout leads to different phenotypes in various cell lines and models, and there is no clear explanation for this variation. Therefore, further investigation is required before MALAT1 could be use as a potential prognositic biomarker (81, 82).

CCAT2 showed extensive effects during proliferation and metastasis in a variety of cancers (47). In fact, patients with high CCAT2 had a lower overall survival and almost twice the risk of death (48).

## LncRNAs in Cancer Therapy

The aberrant expression of lncRNAs and their involvement in diverse cellular processes make them possible targets for cancer therapy. Clinical studies have demonstrated the importance of studying the mechanisms of lncRNA. BC-819, a plasmid containing the promoter of H19 and coding sequence of diphtheria toxin, has been applied in clinical trials of bladder, pancreatic and ovarian cancer (83, 84). The H19 promoter allows diphtheria toxin to be specifically expressed in tumor tissues. Thus, BC-819 can effectively ablate tumors, reduce tumor growth, prolong recurrence time, and has low local toxicity (83–85). We can also learn about the prospect of lncRNAs from patent applications (86). For example, an inhibitor of LINC01212 is used to treat melanoma (US2016271163). What's more, lncMyoD, acts directly as functional element on IMP1 and IMP2 for sarcoma therapy (WO2015020960). Furthermore, although lncRNAs are considered with no protein-coding ability, some special lncRNAs can be translated into micropeptides (87). In fact, some patents utilized these polypeptides for antibodie design in cancer diagnosis and treatment, such as lncRNA-6585 and its antibody in cervical cancers (CN109337903A).

LncRNAs and their loci can be targeted for the design and synthesis of specific nucleic acid sequences in therapy, such as CRISPR/Cas9 design, small interfering RNA (siRNA) and antisense oligonucleotides (ASOs). However, unlike mRNA, most lncRNAs are located in the nucleus and have high-level structure (88, 89). Oligonucleotide drugs must enter cells and bind to their target RNA to function, which raises challenges for drug delivery and intrinsic affinity (90). To address these issues, a common approach is to modify the sequence of oligonucleotides, and the development of nanomedicine to improve drug delivery.

- CRISPR/Cas9 silencing of NEAT1 or MALAT1 was reported to inhibit metastasis of cancer cells (91). A patent used CRISPR/Cas9 to silence UCA1 inhibited the growth of cancer cells (CN106399306B). However, due to the overlap of loci, CRISPR/Cas9 cannot be applied to the silencing of all lncRNAs. In a genome-wide study including 15,929 lncRNA loci, only 38% were successfully silenced as expected, while the remaining had a severe negative impact on the expression of neighboring genes (91).
- Both of siRNA and ASOs can effectively and specifically silence the expression of target genes, making them essential tools for research and clinical uses. Despite some challenges, the progress of siRNAs as therapy drugs has evolved from pre-design to clinical trials (92). Recently, a siRNA targeting DDX11-AS1 has been patented in liver cancer (CN108546702A). Compare to siRNA, ASOs enter the nucleus more efficiently and bind to precursor RNAs near the intron and exon junction, affecting the alternative splicing process (93).
- Methods for affinity and delivery improvement: Affinity of oligonucleotides can be improved by constructing aptamers (94). They can also be modified to reduce nuclease degradation and increase their internal affinity (95, 96). For delivery, one effective approach is to construct drug carriers, such as in the form of gold nanoparticles (97).

## Discussion

In recent years, many studies have been devoted to lncRNAs in cancer progression and treatment. Due to their highly specific expression and diverse functions, lncRNAs hold great promise for cancer diagnosis, prognosis and therapy. To the best of our knowledge, however, the sole lncRNA that has been approved in clinical use is PCA3 in the diagnosis of PCa. Although multiple lncRNAs have been extensively investigated in clinical trials or have been patented, their applications still have a long way to go. Here we review potential lncRNAs that could be considered as biomarkers or therapeutic targets and discuss some of the issues that deserve special consideration.

First, the actual mechanisms by which lncRNA act are not fully understood. Indeed, the developing of oligonucleotide drug Genasense can be served as an informative case. Due to the lack of in-depth understanding of the mechanism, the development of Genasense failed, revealing the importance of understanding the mechanism in drug development (93). Second, the low conservation of lncRNAs, some of which are expressed only in primates, makes it difficult to establish universal experimental models (98). For the majority of lncRNAs, we have yet to establish a suitable animal model, which is essential for understanding the functions better. Third, although some experiments have been conducted on the applications of lncRNAs, they are not very reliable due to the small sample size. Moreover, for therapeutic targets, it is important to study whether the dysregulated expression of lncRNAs is the cause or a result of cancer.

Although there are many challenges, the prospects and clinical significance of lncRNAs cannot be overlooked in the long run. A distinctive feature of lncRNAs is their high specificity in tumor tissues and cells, making it possible for them to be specific and accurate biomarkers (99). In addition, abnormally expressed lncRNAs can be extracted non-invasively, showing great potential to be more economical and less harmful. Compared to protein-based anti-tumor drugs, lncRNA are more refined and less toxic, and the low expression of lncRNA means that only a small amount of inhibitors are needed to make a difference (16). Besides, bioinformatics and computational tools provide new opportunities for lncRNA biomarker development (100). However, due to the lack of experimental evidence and no further clinical validation, we do not discuss it here. Although there are currently no lncRNA-based oncology drugs, drugs targeting lncRNAs in other diseases will provide useful clinical insights.

In conclusion, the intensive study of lncRNAs has brought new hope for the diagnosis and treatment of cancer. Although limitations exist, such as mechanisms, conservative, and animal models, the successful application of PCA3 is a great source of inspiration and impetus for clinical research on lncRNAs. A comprehensive understanding of lncRNA's expression, structure, and mechanisms will help to open up a new intervention, identifying novel and sensitive biomarkers and therapeutic targets.

## Author Contributions

YQ and LS participated in the design of the study, prepared table, figure, and wrote the manuscript. LS and ZL conceived the study and wrote the manuscript. All authors contributed to the article and approved the submitted version.

## Conflict of Interest

The authors declare that the research was conducted in the absence of any commercial or financial relationships that could be construed as a potential conflict of interest.

## Abbreviations

LncRNA: Long Non-coding RNA
ncRNA: Non-coding RNA
miRNA: MicroRNA
PCA3: Prostate Cancer Antigen 3
PCa: Prostate Cancer
ceRNA: Competing Endogenous RNA
PSA: Prostate-specific Antigen
AUC: Area Under the Curve
EGRF: Epidermal Growth Factor Receptor
GC: Gastric Cancer
SNPs: Single Nucleotide Polymorphisms
CRC: Colorectal Cancer
OS: Overall Survival
TCC: Bladder Transitional Cell Carcinoma
EMT: Epithelial to Mesenchymal Transition
HCC: Hepatocellular Carcinoma.
